# ‘What Does Donor Mean to a Four‐Year‐Old?’: Initial Insights into Young Children's Perspectives in Solo Mother Families

**DOI:** 10.1111/chso.12181

**Published:** 2016-08-23

**Authors:** Sophie Zadeh, Tabitha Freeman, Susan Golombok

**Affiliations:** ^1^ Centre for Family ResearchUniversity of CambridgeCambridgeUK

**Keywords:** children's perspectives, donor conception, father absence, solo mother

## Abstract

This study reports on the questions, thoughts and feelings of children aged 4–9 conceived by donor insemination to single mothers. Fifty‐one mothers and 47 children from the same families were each administered a semi‐structured interview. Mothers generally reported that while children either lack understanding, or have not yet been told, about their donor conception, they may be thinking and talking about the absence of a father. Most children did not mention either donor conception or father absence and reported positive feelings about their families and friendships. Possible explanations for the discrepancy between mothers’ and children's reports are discussed.

## Introduction

Of the eight million families with dependent children in the UK today, two million are headed by single parents, most of whom are single mothers (Office for National Statistics, 2015). Despite their increasing prevalence, public and political discourse remains characterised by concerns about the well‐being of children raised by mothers alone (Golombok, 2015). Coupled with this discourse is a growing acknowledgement on the part of researchers and policy‐makers that children's perspectives on family life are worth listening to (Dunn, 2008). However, little is yet known about the perspectives of very young children in single‐mother families created by donor insemination (DI).

In the earliest studies of children's perspectives on family life (Piaget, 1928), it was recognised that children offer different definitions of family at different stages in their development. Although this is now generally accepted (Anyan and Pryor, 2002; Gilby and Pederson, 1982; Morrow, 1998; O'Brien and others, 1996), the criteria upon which children at different developmental stages define who is — and is not — family is less well established, with evidence that young children draw upon biological relatedness (Roe and others, 2006), frequency of contact (Perlesz and others, 2006) and affection (Mason and Tipper, 2008) in their descriptions of what family means to them.

The study of young donor‐conceived children's perceptions of family life — in which there is an immediate biological relative who is not resident — offers an opportunity for greater understanding of this developmental trajectory. Much of the research in this area has been conducted with young children in two‐parent families. At ages 4 and 5, children raised by two mothers have been shown to be able to explain that their families are without a father, and to describe the basic features of their donor conception (DC; Brewaeys and others, 1997; Malmquist and others, 2014), yet at ages 6–10 may not view the topic as salient to their everyday experiences (Van Parys and others, 2015). Donor‐conceived children in heterosexual two‐parent families have been found to have few questions when told about their conception in infancy (Lalos and others, 2007), and to understand little at age 7 (Blake and others, 2010), but by age 10, most demonstrate a basic understanding, and range in their responses from curiosity to no interest in the donor (Blake and others, 2014).

Parents’ reports indicate a similar trend, with most children in heterosexual two‐parent families being described as either interested in or neutral about their DC (Rumball and Adair, 1999), although in one study a minority were reported to have responded negatively to this information (Mac Dougall and others, 2007). More recently, adolescent and adult offspring's retrospective accounts of their initial responses to being told have indicated that age plays a role in determining how information about DC is received, with those told later in life more likely to report negative feelings (Freeman and Golombok, 2012; Hertz and others, 2013; Jadva and others, 2009). Differences according to family type have also been identified, with those in single‐parent families less likely to report feeling confused when first told (Nelson and others, 2013), and more likely to be curious about the donor (Beeson and others, 2011; Scheib and others, 2005).

However, with one notable exception (Nixon and others, 2015), the majority of research investigating young children's perspectives in single‐parent families has focussed on families resulting from divorce or bereavement (Castrén and Widmer, 2015; Dunn, 2008; Nickman and others, 1998; Perry and others, 2004). Scholars adopting a psychodynamic perspective have nevertheless suggested that from a very young age, children conceived by assisted reproduction to single parents are acutely aware of the fact that their families do not generally resemble those of their peers (Ehrensaft, 2000; Moskowitz, 2010). A longitudinal study based on mothers’ reports suggested that in infancy (Landau and Weissenberg, 2010) and at age 7 (Weissenberg and Landau, 2012), donor‐conceived children raised by single mothers have questions about both father absence and DC. In line with Malmquist and others's (2014) study of children in two‐mother families, this study aims to shed further light on these issues by conceptually separating father absence from DC and by involving both mothers and children in the research process.

## Materials and methods

### Participants

Participants were recruited through the UK's largest fertility clinic offering DI to single women. Fifty‐one mothers agreed to take part, giving a response rate of 85 per cent. All mothers had at least one child conceived via DI. Four mothers (8%) had twins. Twenty‐nine (57%) had told their child about their DC, 8 (16%) mothers had ‘partially disclosed’ (i.e. discussed fertility treatment without mentioning donor sperm), and 14 (27%) had not told their children about any aspect of their conception, although 12 (24%) of those mothers planned to do so. Findings related to mothers’ disclosure decisions (Freeman and others, in press) and their thoughts and feelings about the donor (Zadeh and others, 2016) and father absence (Zadeh and others, 2013) are reported elsewhere.

Within these families, 47 children were asked and agreed to be interviewed, 25 (53%) of whom were girls and 22 (47%) were boys. Children's ages ranged from 4 to 9 years (mean = 5.7, SD = 1.65).

### Procedure

Approval for the study was obtained from the University of Cambridge Psychology Research Ethics Committee. Mothers and children took part in a semi‐structured interview on their own at home. Interviews were administered by a research psychologist trained in the study techniques and well aware of the issues specific to conducting ethical research with children. Children were told that researchers were conducting a study of family life and would like to learn about their family, friendships and school experiences. Each participant was reminded that their responses would be regarded as confidential and that they could terminate their participation in all or part of the study at any time; such information was conveyed to children in an age‐appropriate manner both prior to and during participation. Mothers, and where possible children, gave written consent to take part, failing which verbal assent was gained. All interviews were audio‐recorded, transcribed and anonymised.

#### Children's interviews

Children were asked to identify family members, and to describe what, if anything, they would like to change about their family circumstances. Children were also asked about their enjoyment of pre‐school or school, their friendships, whether they had been teased, and if so, about what. Given the brevity of the data obtained, children's responses were coded in the following manner: *family members* was rated as mother, siblings, grandparents, other relatives, donor, donor siblings and pets; *family change* was rated as a wish for structural change, non‐structural change or no change; *enjoyment of pre‐school or school* was rated as none, mixed, mostly or a great deal; *reason for teasing* was rated as family type, other reason or trivial.

#### Mothers’ interviews

Mothers were asked about their children's feelings about growing up without a father in the home, whether their children had expressed feeling different to other children because of their family circumstances and whether they had experienced teasing or bullying because of this. Mothers who had told their children about their DC were asked whether they felt their children understood information about their DC, whether they perceived their children to have any thoughts or feelings about their DC and whether their children had expressed any interest in the donor.

The data obtained from mothers were rated according to the following codes: *child's feelings about father absence* was rated as negative, neutral/indifferent, mixed/ambivalent, positive or not sure; *child's feelings about DC* was rated as negative, neutral/indifferent, mixed/ambivalent, positive or not sure; *child's interest in the donor* was rated as no interest, interest in identity of donor, interest in meeting donor or not sure.

Mothers’ responses were also analysed qualitatively using thematic analysis (Braun and Clarke, 2006). Transcripts were first open‐coded line‐by‐line, and a total of 19 codes across interview scripts were generated. All codes and corresponding text segments were abstracted from the full text and re‐read. Codes were then grouped into higher level conceptual themes, producing seven subthemes relating to two main themes. Regular peer debriefing (Flick, 2014) strengthened confidence in the findings.

## Results

### Descriptive statistics

When asked who is in their family, most children identified their mother (35, 74%). Some mentioned siblings (17, 36%), grandparents (23, 49%), other biological relatives, such as uncles (13, 28%), aunts (12, 26%), cousins (11, 23%) and pets (6, 13%). Two children (4%) described their donor as family, and one (2%) mentioned donor siblings.

When asked about their enjoyment of pre‐school or school, several children (19, 41%) reported high levels of enjoyment, and nine (20%) stated that they found it mostly enjoyable. Fifteen children (33%) reported having mixed feelings and three (6%) said they had no enjoyment in school. Of the 43 children who answered the question about friendships, all reported having at least one friend at pre‐school or school. Around half of the children (22, 51%) named five or more friends.

Of the 37 children who answered the question about changing their family circumstances, 19 (51%) said they would keep their family as it is, and 18 (49%) reported that they would like to make changes. Several (14, 38%) children's changes were non‐structural, referring, for example, to the acquisition of family possessions (a new TV, computer), to changes to particular family members’ character traits (sense of direction, fashion or humour), or to activities not specific to family per se (birthdays, days out). Of the four children (11%) who reported wanting to make changes to family structure, two (5%) wanted pets, one (3%) wanted his grandmother to act as primary caregiver and one (3%) wanted his donor to be involved in family life.

Of the 41 children who answered the question about teasing, 26 (63%) had not been teased at school and 15 (37%) reported having been teased. Most children described this teasing as trivial (14, 34%), although one child (3%) reported having been teased because of her family type.

When asked about their children's feelings regarding father absence, several mothers (20, 39%) reported a neutral or indifferent response. Some (14, 27%) reported that their children had mixed or ambivalent feelings, and others were unsure of how their children felt (11, 22%). A minority of mothers stated that their children felt either negatively (4, 8%) or positively (2, 4%) about growing up without a father in the home. No mothers reported that their children had experienced teasing or bullying because of their family composition.

Of those mothers who had told their children about their DC, 11 (37%) reported that their children were neutral or indifferent about being donor conceived. Eight mothers (28%) stated that their children had mixed or ambivalent feelings, two (7%) reported that their children felt positively and the remainder (8, 28%) were unsure. When asked about the donor, most mothers (20, 69%) reported that their children had no interest, although 6 (21%) stated that their children would like to meet him.

### Qualitative analysis

The most prevalent theme throughout mothers’ reports related to ‘daddy discussions’, with the majority of mothers having had conversations with their children about father absence, irrespective of whether or not they had currently disclosed about DC. Within this theme, three subthemes were identified: ‘discussions about dad — not donor’, ‘prompts for parent–child discussions’ and ‘fantasies about fathers’. A second theme, ‘donor concepts’, was less prevalent overall, but was identified across the responses of mothers who had told their children about their DC. Subthemes within this theme were ‘limited understanding about donor’, ‘fantasies about donor’, ‘curiosity about donor’ and ‘children's disclosure to others’.

#### Daddy discussions

##### Discussions about dad — not donor

Most mothers explained that conversations with their children about father absence were much more frequent than those about the donor. Several responded to questions about their child's understanding of, and feelings towards, the donor with information about their child's feelings about father absence:

They don't talk about the donor. They talk about a father figure and ‘You need to go out and find somebody to be a dad to us’…They don't talk about the biological father. They haven't made that connection. (Mother of seven‐year‐old girls told about DC)

I'm gonna separate donor conceived from not having a dad because I don't know at this age if that has any kind of meaning. What does donor mean to a four‐year‐old?…He knows that he does have a dad that is not part of our lives…I think that's the issue that he's aware of as opposed to having a donor. (Mother of four‐year‐old boy told about DC)

Mothers generally reported that their children had asked about their father and that their children had initiated discussions about this, with some questioning the reason for father absence, and others expressing their preference for a father. The age at which children introduced these conversations and their frequency varied across reports:

I quite distinctly remember…when he was about 2 and a half…his language was quite baby‐fied at that stage, but [he was] saying to me, ‘Oh, she's got a daddy…he's got a daddy, and she's got a daddy and I haven't’, and I just said ‘That's right’, and he said ‘Why?’ (Mother of seven‐year‐old boy told about DC)

It doesn't really [come up now]…A year ago it would be like, ‘I don't have a daddy’…almost fact checking, you know, that that was it. (Mother of four‐year‐old girl partially told about DC)

As above, some mothers explained that the prevalence of their child's questions about the absence of a father had diminished over time. Others explained that their children had made a point of specifying their need for a father with regard to the tasks that he might undertake:

Lately he's been talking about it, mainly because he wanted to improve his strength, and he thinks having a father to play with and to take him to things would improve his strength. (Mother of nine‐year‐old boy partially told about DC)

Sometimes she says, ‘Mummy, it would be good if we had a daddy, then the daddy could cuddle [sibling] and you could cuddle me. (Mother of four‐year‐old‐girl not told about DC)

##### Prompts for parent–child discussions

Several mothers explained that conversations about father absence were initiated by their children often at unexpected times:

He's got to the stage where he has said, ‘Oh I really want a daddy’. He's said that quite a few times and he'll say it quite out of the blue. (Mother of four‐year‐old boy not told about DC)

She really took me by surprise when she said it … We were driving in the car and just completely out of the blue she said ‘Why do I have to only have a mummy?’ (Mother of five‐year‐old girl told about DC)

Many mothers also described how certain social encounters, such as being at school, served as prompts for children's questions:

Something will happen at school and they'll start talking about ‘Why don't we have a dad?’, and that sort of thing … I think they're looking at the family unit and realising that the majority of the other children they know come in a different package … there's only one other in their class without a dad. (Mother of seven‐year‐old girls told about DC)

Relatedly, some mothers reported that their children asked questions about father absence following specific, and sometimes challenging, encounters with other children. Questions from other children were described as being met with different responses:

I think the first time it ever actually came up in a conversation he was about 3 and a half, maybe 3, and a friend of his who was about 9 months older, asked him ‘Where is your dad, have you got a dad?’. And I could see him thinking ‘Hold on a second, I don't know’. (Mother of seven‐year‐old boy told about DC)

A little girl his age said to him, ‘Where's your dad?’…he turned around and said, ‘I don't have one’. And I was looking at him, and he said it really blasé…he was so matter of fact about it … it was like, ‘Well I don't have any sweets on me at the moment’. (Mother of four‐year‐old boy told about DC)

Some mothers explained that specific school activities such as whole‐class games and father's day craft sessions had prompted mother–child discussions about father absence. As in children's responses to other children's questions, their responses to specific school activities were also varied, with some children responding somewhat negatively:

She only gets bothered, really, when it's coming up for father's day when they're doing stuff at school…sometimes she will say ‘I'm not special because I haven't got a dad’. (Mother of six‐year‐old girl told about DC)

A minority of mothers also reported that questions and comments from their children were often preceded by them having spent time with fathers in other families:

They say they want a daddy, when they see the daddies of their other friends they keep on mentioning it. Yes, that they would like a daddy. (Mother of five‐year‐old girls told about DC)

She does mention daddy, like [other] people's daddy and ‘why haven't I got a daddy?’ (Mother of four‐year‐old girl not told about DC)

##### Fantasies about fathers

Several mothers also explained that they had experienced, overheard or otherwise been told about their children's fantasies involving fathers:

I remember him once saying to me ‘My daddy works really far away in a different country’. (Mother of six‐year‐old boy told about DC)

She told another child at playgroup once that her dad was dead, so I don't know if she was trying that out as some sort of story [or] identity. (Mother of four‐year‐old girl told about DC)

Most mothers suggested that these may have been prompted by specific social experiences, and some described having directly discussed them with their children:

She's got a pretend dad, she's got a pretend brother, sister, cat, dog…occasionally I'd test her out a little bit and say ‘I wish he'd come round and help around the house’ or something, and she'd say ‘Mummy, he's only pretend’ [laughs]. (Mother of six‐year‐old girl told about DC)

#### Donor concepts

##### Limited understanding

The majority of mothers who had made their children aware of their DC reported that their children's understanding was limited:

I don't even think he knows what it means … he thinks all babies are born through caesarean. He doesn't know the difference between girls and boys at the moment. (Mother of four‐year‐old boy told about DC)

I think there is an element of not really understanding that he's nominally her father but he's not involved … I think she struggles to understand that, because there's a daddy‐shaped space in people's lives. (Mother of seven‐year‐old girl told about DC)

One mother suggested that her child's limited understanding about the donor was clear from the words used to describe him:

She used to think I was saying donut, I think, at the beginning, and we used to laugh whether it was a chocolate one or a jam one. And I said ‘No, don‐or, don‐or’, and we'd explain about a kidney donor or a blood donor, well this was a seed donor. (Mother of six‐year‐old girl told about DC)

##### Fantasies about the donor

A minority of mothers described conversations with their children that detailed fantasies about the donor and their conception. Some attributed this to a lack of understanding about, or interest in, the process:

So if I say, ‘Well remember mummy said I went to the doctor, because I really wanted to have you, and mummy didn't have a husband’, and all that stuff, and he's listening, and then he'll add this little fantasy thing, ‘Then did this happen’ and ‘Did that’ [laughs]…So he's not quite focussing. (Mother of six‐year‐old boy partially told about DC)

Others explained that their children's fantasies about the donor were more elaborate, and part of conversations that happened both inside and outside of home:

He does make up stories. He tells me his donor dad was there when he was born… but I didn't know what he looked like as I didn't see him, and there's occasional stories at other times. I told him that he has several half‐siblings, and for a while there was…a girl in Russia who was his half‐sister…so he does kind of fantasise around it. (Mother of four‐year‐old boy told about DC)

She'd been going around telling people at school that her donor daddy was 7 foot tall, which he's not … I did wonder whether that was ‘Oh, my donor daddy's bigger than your real daddy!’ (Mother of six‐year‐old girl told about DC)

##### Curiosity about the donor

A minority of mothers also described that their children were currently curious about the donor:

She calls him her daddy. She'd love to know him now if she could. (Mother of six‐year‐old girl told about DC)

He says that one time he'll go [abroad] so he can see his dad…I think it's particularly if children are talking about their dads, particularly at school he might say it. I don't think it's a real drive in him or anything. (Mother of eight‐year‐old boy told about DC)

Other mothers suggested that their children's curiosity was less about the donor and more about other information they had learned from conversations about their conception:

I was explaining that we know that the sperm donor is a very kind man, and his comeback was, ‘How do you know? You've never met him’…He also said, ‘If he was so kind and nice, why can't you just marry him?’ (Mother of seven‐year‐old boy told about DC)

He was more interested in the doctor [laughs] what happened to the doctor! (Mother of six‐year‐old boy told about DC)

##### Children's disclosure to others

Finally, a minority of mothers’ reports detailed unanticipated occasions wherein children had themselves told others about their conception:

We went to a local park here and [one of the] typical ladies…started chatting with [child], and she said, ‘I bet your daddy really dotes on you’, and [child] turned to her and goes, ‘No, I've got a donor daddy’, and the face, I mean I just wish I had a picture [laughs]. (Mother of four‐year‐old boy told about DC)

We were on the underground. This drunkard man came up to us … and said, ‘Don't you ever lose touch with your father’. He obviously had children problems. And she said, ‘My dad's a donor’, like that, and the whole carriage heard and swung round and me and my mum just chuckled to each other. It was brilliant [laughs]. (Mother of six‐year‐old girl told about DC)

## Discussion

The findings of this study offer an initial insight into what young donor‐conceived children raised by solo mothers may be thinking and talking about, both within the family home and outside of it. From mothers’ reports, it seems that children aged 4–9 are more focussed on father absence than DC. As in earlier research (Landau and Weissenberg, 2010; Weissenberg and Landau, 2012), questions and comments from children about the absence of a father were widely reported by mothers, who generally stressed the significance of social context in shaping their children's thoughts and feelings about family life. Of course, the idea that the thoughts and feelings of children — not least children who are donor conceived — may be shaped by their social experiences is neither new nor controversial. However, and although not without exception (Freeman and others, in press), the finding that young children in solo mother families generally appear to be more focussed on father absence than on DC is an important consideration for practitioners and policy‐makers working in the field. While there is now a wealth of resources for talking to young children about DC (Mendell and Sarles, 2010), findings indicate that comprehensive and widely distributed resources to support single parents in answering their donor‐conceived children's questions about family life in general — and the absence of a second parent in particular — may still be lacking. Given that several of the mothers in this study had not yet told their children about their donor conception, and some were unsure about whether or not to do so, it is likely that resources focussed on the issue of father absence, rather than the donor, would be welcomed.

Children's reports of their experiences were generally favourable, and the majority described their families and friends in a positive manner. These findings are of particular interest when considered against the broader literature on children in divorced single‐parent families, who have been found to have less extensive friendship networks than their two‐parent counterparts (Dunn, 2008). It is worth noting, however, that in the present study one child reported having been teased about father absence. Moreover, several mothers reported that their children were unable to fully participate in school activities because of their family circumstances and that they had been asked by their peers to explain their families. Similar to findings of research with donor‐conceived children in two‐mother families (Raes and others, 2015; Van Parys and others, 2015; Vanfraussen and others, 2002), it seems that the social world of the children in this study may be characterised by assumptions about what families are and/or ought to be like. Although the children who participated were found to be generally well‐adjusted (Golombok and others, 2016), amendments to the school syllabus that emphasise family diversity, and the implementation of teaching resources about single‐parent families akin to those developed about same‐sex parents (Guasp, 2010), may now be helpful.

Some of the more complex findings relate to the apparent discrepancy between mothers’ reports of children's questions, thoughts and feelings, and children's own reports, the latter of which did not, for the most part, mention either DC or father absence. There are several possible explanations for this. First, only around half of the mothers in this study had told their children about their DC; the remainder of the children did not yet have access to this information, and so would not have raised it with researchers. However, it is worth noting that of the total number interviewed, only two children made reference to their donor and only one child discussed father absence. This latter aspect of family life, at least, would have seemingly been apparent to them all.

A second interpretation relates to the study's main limitations. First, mothers of children distressed by father absence and/or DC may have been less inclined to participate in the study. Second, no questions about fathers were asked of the children who did participate. Given the sensitive nature of the issues under study, it was thought to be inappropriate to ask children such direct questions, and, as in other research (Malmquist and others, 2014), interviews therefore aimed to elicit children's spontaneous reports about family life. As a result, the findings may have been influenced by methodological shortcomings and thus under‐reported children's genuine thoughts and feelings. Indeed, given that mothers described children's detailed fantasies about fathers — and sometimes donors — and given that research has shown a similar trend among adopted children regarding their birth parents (Brodzinsky, 2011), it may be that the children in this study did not raise these topics with researchers because they would have felt uncomfortable discussing them with unfamiliar adults. As alternative methods such as play‐based tasks and drawings have generated different findings from donor‐conceived children in other family types (Perry and others, 2004; Raes and others, 2015), it is recommended that researchers now think more creatively about how to engage young donor‐conceived children in research about topics that may be difficult to discuss directly.

Conversely, it has been suggested that donor‐conceived children might express indifference about their conception because of a sense of loyalty to their families, rather than because they are genuinely disinterested (Vanfraussen and others, 2001). There is also the possibility that most children did not discuss father absence or DC because these issues are simply not at the forefront of their thoughts; as identified by Blake and others (2014), it may well be that children's everyday experiences are such that DC is considered to be less interesting than other aspects of their lives. Indeed, it is important that different family members’ perspectives on family life are given equal consideration by researchers (Harden and others, 2010), and that should these perspectives differ, they are nevertheless considered to be valid indicators of participants’ individual experiences.

Although the findings of this investigation paint a rather complex picture, this study serves as an important initial insight into the thoughts, feelings and experiences of young children conceived by DI to single mothers. Given mothers’ reports that conversations about father absence are frequent during early childhood, it is recommended that resources for parents and teachers that focus on this issue are developed. The findings of this study attest to the difficulties of gaining comprehensive verbal accounts from young children, yet the highlighted discrepancy between mothers’ and children's reports serves as a reminder of the necessity of obtaining children's perspectives. Further research that adopts an alternative methodological approach is strongly encouraged.

## Funding

This study was funded by the Wellcome Trust (097857/Z/11/Z).
